# Revisiting Antiplatelet Therapy in Acute Carotid Tandem Lesions

**DOI:** 10.3390/jcm15093195

**Published:** 2026-04-22

**Authors:** Matija Zupan, Lara Straus, Pawel Kermer, Panagiotis Papanagiotou, Senta Frol

**Affiliations:** 1 Department of Vascular Neurology, University Medical Centre Ljubljana, 1000 Ljubljana, Slovenia; matija.zupan@kclj.si (M.Z.); lara.straus@kclj.si (L.S.); 2 Faculty of Medicine, University of Ljubljana, 1000 Ljubljana, Slovenia; 3 Department of Neurology, Nordwest-Krankenhaus Sanderbusch, Friesland Kliniken GmbH, 26452 Sande, Germany; pkermer@gwdg.de; 4 Department of Neurology, University Medical Center Göttingen, 37075 Göttingen, Germany; 5 Clinic for Diagnostic and Interventional Neuroradiology, Klinikum Bremen Mitte, 28211 Bremen, Germany; nrpap@me.com; 6 Department of Radiology, Aretaieion University Hospital, National and Kapodistrian University of Athens, 10679 Athens, Greece

**Keywords:** acute carotid tandem lesions, acute ischemic stroke, antiplatelet therapy, cangrelor, glycoprotein IIb/IIIa inhibitors

## Abstract

**Background/Objectives**: Acute carotid tandem lesions (TLs), defined by concurrent cervical internal carotid artery (ICA) stenosis or occlusion and intracranial large vessel occlusion, occur in 10–20% of patients undergoing mechanical thrombectomy (MT) for acute ischemic stroke (AIS). Optimal periprocedural antiplatelet management during emergent carotid artery stenting (eCAS) remains uncertain, particularly regarding the balance between preventing stent thrombosis and avoiding hemorrhagic complications. **Methods**: A narrative review was conducted using PubMed and Scopus (until 6 March 2026) to identify English-language studies evaluating antiplatelet therapies during eCAS for TLs. We included seven real-world studies and registry analyses. Data on study design, patient characteristics, procedural strategies, angiographic results, functional outcomes, and safety metrics were extracted. **Results**: No randomized controlled trials (RCTs) were identified. The available evidence is derived exclusively from observational studies. Across these cohorts, glycoprotein IIb/IIIa inhibitors (GPIs), particularly tirofiban, were generally associated with lower rates of in-stent thrombosis and higher reperfusion success, with symptomatic intracranial hemorrhage (sICH) rates that appeared comparable to or lower than those reported with acetylsalicylic acid (ASA). Cangrelor, an intravenous (IV) P2Y_12_ inhibitor, was associated with improved stent patency and increased likelihood of complete reperfusion, although reported effects on clinical outcomes were inconsistent when compared with GPIs or ASA. Aside from abciximab, potent IV antiplatelet agents did not consistently show an increased sICH signal. Oral dual antiplatelet therapy was also associated with improved technical outcomes without a clear excess in bleeding complications. **Conclusions**: Current real-world observational data suggest that rapid-acting IV antiplatelet agents—particularly GPIs and, increasingly, cangrelor—may represent feasible periprocedural options during eCAS for TLs, with potential benefits for technical success and no consistent evidence of increased hemorrhagic risk. However, interpretation is limited by study heterogeneity and non-randomized designs. The absence of RCTs highlights the need for prospective comparative studies and standardized periprocedural antiplatelet protocols.

## 1. Introduction

Acute carotid tandem lesions (TLs) refer to the simultaneous presence of high-grade stenosis or acute occlusion of the cervical internal carotid artery (ICA) and thromboembolic occlusion of the intracranial terminal ICA or its branches, usually the middle cerebral artery (MCA), in the setting of acute ischemic stroke (AIS) [1,2]. TLs are encountered in approximately 10–20% of large vessel occlusion (LVO) cases undergoing mechanical thrombectomy (MT) [1,2,3], and their presence is generally associated with worse and up to 50% lethal outcomes [1,3]. Stroke patients with polyvascular atherothrombotic disease showed higher rates of vascular recurrence and a stronger association with inflammatory markers [4]. Intravenous thrombolysis (IVT) is less effective in TLs compared with an isolated intracranial thromboembolic occlusion [1,3].

It has been shown that emergent carotid angioplasty with stenting (eCAS) is associated with a better clinical outcome compared to balloon angioplasty in treating extracranial lesions [5,6]. However, many unanswered questions remain regarding peri- and postprocedural antiplatelet treatment in these patients. For example, the HERMES Collaboration pooled individual data from five seminal MT trials, yet only ~10% had TLs. No significant interaction was observed between treatment effect and TL status for the 90-day modified Rankin Scale (mRS) outcome [7,8,9,10,11,12]. While this study supported the use of MT in TL patients, it provided little guidance on optimal antiplatelet regimens or endovascular techniques [3]. Likewise, the AURORA meta-analysis evaluated data from six late-window MT trials, but only four included TL patients, limiting definitive conclusions [13].

Furthermore, the reported risk of stent thrombosis with re-occlusion of ICA is substantially high, with rates from 14.5% [14] up to 45% [15]. The highest risk of stent thrombosis was observed during the first 24 h [14], showing a trend towards worse 90-day functional outcomes [16]. Independent predictors for carotid re-occlusion included the degree of recanalization, the presence of pial collaterals, and the use of general anesthesia during MT. By contrast, eCAS was independently associated with lower odds of re-occlusion [16].

Antiplatelet agents are necessary in peri-interventional care to avoid stent thrombosis [17]. Patients already receiving antiplatelets are likely to be at low risk of stent thrombosis; hence an aggressive antiplatelet strategy appears optional [17]. The Society of Neurointerventional Surgery 2023 guidelines recommend dual antiplatelet therapy (DAPT) to be initiated before carotid artery stenting and be continued for at least three months thereafter (Class IIa, Level B-R) [18]. These guidelines state that it may be reasonable to administer a loading dose of intravenous (IV) glycoprotein IIb/IIIa inhibitor (GPI) or P2Y_12_-inhibitor followed by maintenance IV infusion to prevent stent thrombosis irrespective of whether the patient has or has not received IVT (Class IIb, Level of Evidence C—Limited Data) [18]. However, there are still no universal guidelines and/or consensus statements on the choice of antiplatelets and their dosing in eCAS for TLs. Thus, heterogeneous antiplatelet regimens are applied globally, such as IV acetylsalicylic acid (ASA), clopidogrel, ticagrelor, GPIs or the more recent IV P2Y_12_ inhibitor cangrelor [17].

The key question remains whether one can avoid acute stent thrombosis by improving the peri-interventional antiplatelet regimen without concomitant increase in symptomatic intracranial hemorrhage (sICH). ASA and clopidogrel inhibit two pivotal pathways of platelet activation with delay. In contrast, GPIs, by direct inhibition of the most abundant receptor on the platelet surface, are highly effective in preventing stent thrombosis, but with increased bleeding risk when given at full dosage [19].

In this narrative review, we provide an update on antiplatelets in acute carotid TLs treated with eCAS.

## 2. Materials and Methods

We searched PubMed and Scopus up to 6 March 2026 for studies reporting antiplatelet use in patients with carotid TLs in the setting of AIS. In addition, we searched references of related letters, reviews and editorials to identify other potentially eligible studies. To be eligible for the present narrative review, the studies had to be published full-text articles in the English language. We screened for the following data: study type, patient characteristics, procedural strategies, primary and secondary outcomes, and safety outcomes.

## 3. Results

We included seven studies, all of which were real-world studies (RWSs) or registry-based analyses; no published randomized controlled trials (RCTs) or meta-analyses specifically comparing antiplatelet strategies during eCAS for TLs were identified (Table 1). The findings are split into several domains: (i) reperfusion success and angiographic outcomes, (ii) carotid stent patency and in-stent thrombosis, (iii) hemorrhagic risk, and (iv) functional outcomes and mortality. Supplementary Table S1 shows details on the timing and dosage of drugs, procedural strategy and stent types utilized in the included studies.

(i) **Reperfusion success and angiographic outcomes**

Across studies, more intensive antiplatelet strategies, particularly IV agents and oral DAPT, were most consistently associated with improved angiographic reperfusion. In the large multicenter analysis by Farooqui et al., antiplatelet strategies were categorized as no antiplatelet therapy, single oral agent, oral DAPT, or IV agents. Among 595 patients undergoing MT for TLs, 119 received no antiplatelet therapy, 134 received a single oral agent, 152 received oral DAPT, and 196 received IV therapy [20]. Antegrade procedural strategy was implemented in the majority of patients across all subgroups. The proportion of patients treated with preceding IVT differed between groups, being lowest in the IV group (38%) and highest in the no-antiplatelet group (54%). While no significant association with sICH was observed, the odds of successful reperfusion were significantly higher with oral DAPT and IV therapy compared with no therapy. Furthermore, the odds of excellent reperfusion (mTICI 2c/3) were significantly higher for cangrelor compared with no antiplatelet therapy [20].

Similarly, Jumaa et al. compared cangrelor with GPIs (tirofiban or eptifibatide) in 63 patients with acute TLs (30 treated with cangrelor and 33 treated with GPIs), without the use of additional oral antiplatelet therapy [21]. Although procedural strategy was not reported, and prior IVT data were not provided (three patients in each group received intra-arterial thrombolysis), cangrelor was associated with a significantly higher rate of complete reperfusion, while the observed reduction in sICH did not reach statistical significance [21].

In the ARTISTA Registry analysis by Medina-Rodriguez et al., 181 patients undergoing eCAS were treated with either ASA (57%) or tirofiban (43%), with 82% of the cohort presenting with TLs [22]. Baseline characteristics were largely comparable, although tirofiban-treated patients more frequently had diabetes, higher Alberta Stroke Program Early CT Score (ASPECTS), and less frequent concomitant intracranial occlusion. The prevailing procedural strategy was retrograde unless cervical ICA stenosis/occlusion prevented it, and preceding IVT rates were comparable between groups. Tirofiban was associated with higher rates of excellent reperfusion (eTICI 2c–3) compared with ASA [22].

(ii) **Carotid stent patency and in-stent thrombosis**

Beyond intracranial reperfusion, carotid stent patency and early in-stent thrombosis represent clinically important outcomes in TL interventions. In ARTISTA, Medina-Rodriguez et al. found tirofiban to be associated with a significantly lower rate of in-stent thrombosis within the first 24 h compared with ASA [22].

Registry analyses from the French ETIS database further explored this theme. Pop et al. included 384 patients with TLs: 91 received cangrelor, 77 GPIs, and 216 received ASA only [23]. While procedural strategy and rates of preceding IVT were not specified in the main text, cangrelor was associated with improved carotid stent patency at day 1 compared with ASA, although this did not translate into statistically significant differences in clinical outcomes. Importantly, no outcome differences were observed between full-dose and low-dose cangrelor [23].

Similarly, Marnat et al. examined 187 patients with atherosclerotic TLs undergoing eCAS and MT, comparing ASA alone (n = 124) to an “aggressive antiplatelet” strategy (n = 63), which included clopidogrel, ticagrelor, a GPI, and/or cangrelor (21 patients received cangrelor alone and two received a combination of cangrelor and a GPI) [24]. Antegrade strategy predominated in both groups. IVT was administered significantly more often in the ASA-only group than in the aggressive antiplatelet group. Although no significant differences were found in overall safety, functional outcomes, mortality, or procedural complications, the aggressive antiplatelet group had a significantly higher rate of carotid stent patency at day 1 [24]. In a subgroup analysis comparing single aggressive antiplatelet therapy (48 patients) with oral DAPT (15 patients), carotid patency remained similarly high (96% vs. 100%, *p* = 0.551), suggesting that both strategies may achieve comparable early stent durability in this selected cohort [24].

(iii) **Hemorrhagic risk**

Hemorrhagic transformation and sICH remain key concerns when combining revascularization therapies with potent antiplatelet regimens, particularly in the context of preceding IVT. Across studies, a consistent increase in sICH was not uniformly observed with IV antiplatelet strategies, although signals differed between agents.

In the large cohort studied by Farooqui et al., no significant association between antiplatelet strategy and sICH was detected, despite differences in preceding IVT rates across treatment categories [20]. In ARTISTA, tirofiban was associated with a significantly lower rate of sICH within the first 24 h compared with ASA, although the tirofiban group differed in baseline characteristics and had less frequent concomitant intracranial occlusion, which may have influenced hemorrhagic risk [22]. Jumaa et al. observed a non-significant reduction in sICH with cangrelor compared with GPIs [21]. In ETIS, Pop et al. did not report an excess hemorrhagic risk with cangrelor relative to ASA or GPIs, although detailed procedural and thrombolysis parameters were not clearly presented in the main text [23]. Likewise, Marnat et al. found no significant safety differences—including sICH—between aggressive antiplatelet regimens and ASA alone, despite IVT being more frequent in the ASA-only group [24].

However, a potential safety concern emerged specifically with abciximab. Delvoye et al. evaluated 60 patients with either TL or isolated extracranial ICA occlusion from a prospective single-center registry; 44 patients (73%) had TL and underwent eCAS, receiving cangrelor, abciximab, or ASA [25]. Interpretation was limited by an unequal distribution of TLs between groups (79% in the ASA group, 50% in the abciximab group, and 78% in the cangrelor group), as well as concomitant administration of other hemostasis-modifying agents, including heparin (in patients without prior IVT) and ASA (administered to 25% of abciximab patients and 67% of cangrelor patients). Preceding IVT rates also differed, ranging from 50% in the abciximab group to 33% in the cangrelor group, and procedural strategy was not specified. Although not statistically significant, abciximab was associated with higher mortality (38%) and higher sICH rates (25%) compared with ASA (mortality 19%, sICH 9%) and cangrelor (mortality 11%, sICH 11%) [25].

This potential signal was echoed in an early retrospective single-center analysis by Heck et al., which included 23 consecutive patients with TL treated with eCAS followed by MT using an antegrade strategy [26]. All patients received 300 mg ASA suppository at procedure start. Twelve patients without prior IVT received IV abciximab, and one additional patient received abciximab after IVT due to intraprocedural stent thrombosis. Intracranial target occlusions were ICA terminus/MCA or M1 MCA in 21 patients, while two had M2 MCA occlusions. Carotid stenting was successful in all cases. Stent thrombosis at 24 h occurred in one of 18 patients, and no delayed thromboses were noted over 24 months. However, five patients (22%) suffered sICH, all within 24 h and fatal. The rate of sICH was higher among abciximab-treated patients (31%) compared with those not receiving abciximab (10%). All patients with sICH were older than the cohort mean, including three aged >80 years. Notably, none of the patients with sICH had received prior IVT [26].

(iv) **Functional outcomes and mortality**

Despite signals suggesting improved reperfusion and stent patency with more aggressive antiplatelet strategies, translation into functional benefit was inconsistent. Farooqui et al. found no significant differences between antiplatelet strategy groups in functional independence or mortality, despite higher odds of reperfusion success with oral DAPT and IV therapy [20]. Similarly, Medina-Rodriguez et al. observed no significant differences in functional outcomes or mortality between tirofiban and ASA, despite improved angiographic outcomes and lower in-stent thrombosis rates with tirofiban [22]. Jumaa et al. also reported no differences in mortality or functional independence between cangrelor and GPI-treated patients, despite higher rates of complete reperfusion with cangrelor [21].

In ETIS, Pop et al. reported that cangrelor was associated with a negative shift in the distribution of mRS scores compared with GPIs, whereas GPIs were associated with higher rates of functional independence compared with ASA [23]. These findings raise the possibility that, within this registry cohort, GPIs may have been linked to more favorable clinical outcomes than either ASA or cangrelor; however, the observational design and potential confounding by indication remain major limitations.

In contrast, Marnat et al. reported no significant differences in overall functional outcomes between ASA-only and aggressive antiplatelet strategies [24]. Yet, in their subgroup analysis comparing single aggressive antiplatelet therapy with oral DAPT, DAPT was associated with higher rates of favorable and excellent 90-day functional outcomes (82% vs. 47%, *p* = 0.036; and 64% vs. 23%, *p* = 0.010, respectively), while sICH rates remained similar [24]. This finding suggests that oral DAPT may potentially confer clinical benefit in selected patients, although subgroup sample sizes were small (48 vs. 15 patients), and residual confounding cannot be excluded.

Finally, Delvoye et al. observed that favorable neurological outcomes were numerically more frequent with cangrelor and ASA compared with abciximab (67% and 58% vs. 38%), while mortality and sICH were also higher in the abciximab group, though none of these comparisons reached statistical significance [25]. Similarly, Heck et al. reported functional independence (mRS ≤ 2) in 52% of patients and a 90-day mortality of 39%, with fatal sICH events contributing substantially to poor outcomes [26].

**Table 1:** Characteristics of the included studies.

Study & Type	Aim	Demographics	Study Groups	Preceding IVT	Primary Outcome(s)	Secondary Outcome(s)	Conclusion
**Farooqui et al., 2025 [20]:** Retrospective multicentric observational cross-sectional study	To determine safety of different APT regimens during eCAS in TLs	595 patients 67 years Female 33% NIHSS 16	No APT: 20% SAPT: 22% DAPT: 25% IV APT: 33%	No APT: 54% SAPT: 44% DAPT: 39% IV APT: 38% ***p* = 0.039**	**sICH (ECASS III)** No APT: 6% SAPT: 4% DAPT: 2% IV APT: 6% *p* = 0.25 SAPT: aOR 0.64, 0.20–2.06, *p* = 0.45 DAPT: aOR 0.35, 0.09–1.43, *p* = 0.15 IV APT: aOR 1.05, 0.39–2.85, *p* = 0.917	**90-day mRS 0–2** SAPT: aOR 1.30, 0.66–2.54, *p* = 0.44 DAPT: aOR 0.91, 0.44–1.86, *p* = 0.79 IV APT: aOR 1.07, 0.50–2.28, *p* = 0.86 **^†^ mTICI 2b–3** SAPT: aOR 0.93, 0.46–1.86, *p* = 0.83 DAPT: aOR 5.85, 2.12–16.14, ***p* = 0.001** IV APT: aOR 2.35, 1.07–5.18, ***p* = 0.034** **90-day mortality** SAPT: aOR 0.52, 0.21–1.26, *p* = 0.146 DAPT: 0.95, 0.36–2.49, *p* = 0.92 IV APT: aOR 0.89, 0.34–2.30, *p* = 0.81 **Sensitivity analysis stratified by different IV APTs** **^†^ mTICI 2c–3** Cangrelor compared with no APTs (aOR 4.41, 1.20–16.28, ***p* = 0.026**)	DAPT and combined IV APTs + DAPT associated with increased odds of successful and excellent reperfusion, without increasing the rate of sICH or mortality
**Pop et al., 2024 [23]:** Sub-analysis of ETIS Registry (prospective, multicenter observational registry)	To determine effectiveness and safety of cangrelor compared with GPIs and ASA monotherapy in TLs treated by eCAS	384 patients	Cangrelor: 24% GPIs: 20% (12% tirofiban, 5% eptifibatide, 4% unspecified) ASA: 56%	Not specified	**90-day functional outcome** (mRS shift analysis: 1-point mRS improvement) **Cangrelor vs. GPIs**: aOR 0.48, 0.25–0.94, ***p* = 0.033** **Cangrelor vs. ASA**: aOR 1.14, 0.63–2.08, *p* = 0.683 **GPIs vs. ASA**: aOR 1.89, 1.03–3.45, ***p* = 0.040**	**90-day functional independence (mRS 0–2**) **Cangrelor vs. GPIs**: aOR 0.70, 0.48–1.02, *p* = 0.062 **Cangrelor vs. ASA**: aOR 1.18, 0.56–2.44, *p* = 0.661 **GPIs vs. ASA**: aOR 2.56, 1.08–6.25, ***p* = 0.033** **Intracranial recanalization status at the end of the procedure (^†^ mTICI 2b/3)** **Cangrelor vs. GPIs**: aOR 0.60, 0.19–1.85, *p* = 0.376 **Cangrelor vs. ASA**: aOR 2.38, 95% CI 0.75–7.69, *p* = 0.144 **GPIs vs. ASA**: aOR 5.26, 0.66–50.00, *p* = 0.118 **Day 1 sICH (ECASS I)** **Cangrelor vs. GPIs**: aOR 0.81, 0.45–1.42, *p* = 0.491 **Cangrelor vs. ASA**: aOR 0.72, 0.27–1.96, *p* = 0.528 **GPIs vs. ASA**: aOR 1.47, 0.54–4.00, *p* = 0.444 **Day 1 carotid stent patency** **Cangrelor vs. GPIs**: aOR 0.86, 0.20–3.72, *p* = 0.837 **Cangrelor vs. ASA**: aOR 4.00, 11.9–14.29, ***p* = 0.025** **GPIs vs. ASA**: aOR 1.89, 0.54–6.67, *p* = 0.320 **90-day mortality** **Cangrelor vs. GPIs**: aOR 1.54, 0.86–2.78, *p* = 0.150 **Cangrelor vs. ASA**: aOR 1.04, 0.49–2.22, *p* = 0.910 **GPIs vs. ASA**: aOR 0.63, 0.24–1.64, *p* = 0.337	Cangrelor with lower odds of good clinical outcomes compared with GPIs; GPIs with higher odds of good clinical outcomes compared with ASA
**Marnat et al., 2023 [24]:** Sub-analysis of ETIS Registry (prospective, multicenter observational registry)	To investigate the safety and efficacy of acute aggressive APTs (GPIs, P2Y_12_ inhibitors) for eCAS in **atherosclerotic** TLs treated with MT compared with ASA	187 patients 67 years Female 35% NIHSS 14	Aggressive APTs: 34% ASA: 66%	ASA: 61% Aggressive APTs: 29%, ***p* < 0.001**	**Carotid stent patency day 1** 97% in aggressive APTs vs. 82% in ASA-only (aOR 17.49, 1.10–277.2, *p* = **0.042**	**90-day functional outcomes** mRS 0–2: 54% aggressive APTs vs. 39% ASA-only (aOR 2.04, 0.81–5.08, *p* = 0.125 **90-day mortality** 13% aggressive APTs vs. 17% ASA-only (aOR 0.55, 0.15–2.00, *p* = 0.369) **Day 1 sICH (ECASS II)** 11% aggressive APTs vs. 8% ASA-only (aOR 1.59, 0.42–5.95, *p* = 0.487)	Aggressive APTs associated with increased likelihood of carotid stent patency on day 1 without increasing risk of sICH and procedural complications
**Medina-Rodriguez et al., 2025 [22]:** Retrospective single-center study of prospective ARTISTA Registry	To evaluate efficacy and safety of IV tirofiban in monotherapy over IV ASA in eCAS + MT in **atherosclerotic** carotid occlusions (**TLs in 82%**)	181 patients 68 years NIHSS 15	ASA: 57% Tirofiban: 43%	ASA: 34% Tirofiban: 30%, *p* = 0.52	**In-stent thrombosis after 24 h** Tirofiban 1.3% vs. ASA 9% (aOR 0.11, 0.01–0.98, ***p* = 0.048**)	**mRS ≤ 2** ASA 52% vs. tirofiban 58% (aOR 1.06, 0.49–2.32, *p* = 0.87) **^†^ eTICI 2c–3** ASA 49% vs. tirofiban 69% (aOR 2.15, 1.12–4.13, ***p* = 0.02**) **sICH (ECASS I)** ASA 12% vs. tirofiban 3% (aOR 0.16, 0.03–0.87, *p* = **0.034**) **90-day mortality** ASA 24% vs. tirofiban 16% (aOR 0.84, 0.35–2.01, *p* = 0.69)	Tirofiban significantly reduced in-stent thrombosis and sICH, but improved rates of excellent reperfusion compared with ASA
**Jumaa et al., 2023 [21]:** Multicentric non-randomized retrospective analysis	To compare the safety profile of low dose IV cangrelor vs. IV GPIs in eCAS in TLs	63 patients 68 years Female 27%	Cangrelor: 48% GPIs: 52%	Only data on IAT: 3% vs. 3%, *p* = 0.981	**sICH (ECASS III)** Cangrelor vs. GPIs: aOR 0.21, 0.02–2.18, *p* = 0.229	**^†^ mTICI 3** Cangrelor 57% vs. GPIs 24%, aOR 5.86, 1.57–26.62, ***p* = 0.013** **90-day 0–2 mRS** Cangrelor 56% vs. GPIs 58%, aOR 2.68, 0.61–13.93, *p* = 0.209 **90-day mortality** Cangrelor 11% vs. GPIs 10%, aOR 0.85, 0.11–7.04, *p* = 0.874	Low dose cangrelor with similar safety and increased rate of complete reperfusion compared with GPIs
**Delvoye et al., 2021 [25]:** Retrospectively selected cases from prospective monocentric registry	Clinical and radiological effects of abciximab, cangrelor and ASA during eCAS (**TLs in 73%; abciximab 50%, cangrelor 78%, ASA 79%**)	60 patients 62 years Female 35% NIHSS 16	Abciximab: 13% Cangrelor: 15% ASA: 72%	Abciximab: 50% Cangrelor: 33% ASA: 54%, *p*-value not stated	**90-day mRS ≤ 2** Abciximab: 38% Cangrelor: 67% ASA: 58%; *p* = 0.72 **ENI at 24 h** Abciximab: 13% Cangrelor: 67% ASA: 48%, *p* = 0.47	**Patency of EC-ICA stent at 24 h** Abciximab: 75% Cangrelor: 63% ASA: 74%, *p* = 0.67 **Reperfusion ^†^ TICI ≥ 2b** Abciximab: 75% Cangrelor: 100% ASA: 91%, *p* = 1.00 **sICH (ECASS III)** Abciximab: 25% Cangrelor: 11% ASA: 9%, *p* = 1.00 **90-day mortality** Abciximab: 38% Cangrelor: 11% ASA: 19%, *p* = 1.00	Cangrelor and ASA with less sICH and higher rate of good clinical outcome compared with abciximab
**Heck et al., 2015 [26]:** Retrospective single-center observational study	To compare their own outcomes and complications with limited published data	23 patients 70 years NIHSS 17	ASA: 100% Abciximab: 57%	ASA-only: 100% Abciximab: none	Whole cohort **^†^ TICI 2b–3:** 74% **^†^ TICI ≥ 2A:** 91% **mRS ≤ 2:** 52% **90-day mortality:** 39% **Stent thrombosis at 24 h:** 1/18, no delayed thromboses during the following 24 months **sICH (SITS-MOST):** 22% (all older than the mean), all within 24 h (all fatal) Abciximab: 31% ASA-only: 10% None with prior IVT		Primary eCAS combined with MT can be an effective treatment in TLs, albeit with a higher sICH incidence, possibly associated with abciximab and advanced patient age

aOR: adjusted odds ratio; APTs: antiplatelets; ARTISTA: A Registry for Thrombectomy In Stroke Therapy from Andalusia; ASA: acetylsalicylic acid; DAPT: oral dual antiplatelet therapy; eCAS: emergent carotid artery stenting; EC-ICA: extracranial internal carotid artery; ECASS I: European Cooperative Acute Stroke Study I; ECASS II: European Cooperative Acute Stroke Study II; ECASS III: European Cooperative Acute Stroke Study III; ENI: early neurological improvement, defined as a drop of at least 8 points of the NIHSS in the first 24 h or a NIHSS of 0–1 at 3 days; eTICI: Expanded Treatment in Cerebral Infarction scale; ETIS: Endovascular Treatment in Ischemic Stroke Registry; FD: flow diverter; GPI: glycoprotein IIb/IIIa inhibitor; IAT: intra-arterial thrombolysis; ICH: intracranial hemorrhage; IV: intravenous; IVT: intravenous thrombolysis; mRS: modified Rankin Scale; MT: mechanical thrombectomy; mTICI: Modified Treatment in Cerebral Infarction scale; NIHSS: National Institutes of Health Stroke Scale; PO: peroral; SAPT: oral single antiplatelet therapy; sICH: symptomatic intracranial hemorrhage; SITS-MOST: Safe Implementation of Thrombolysis in Stroke-Monitoring Study; TICI: Thrombolysis in Cerebral Infarction scale; TL: tandem lesion. ^†^ Reperfusion outcomes across the included studies were reported using different thresholds of the modified or expanded Treatment in Cerebral Infarction scales (mTICI/eTICI), including mTICI ≥ 2b, mTICI 2b–3, mTICI 2c–3, mTICI 3, and eTICI 2c–3. These metrics describe related but not identical degrees of reperfusion. Traditionally, mTICI ≥ 2b (or 2b–3) is used to define successful reperfusion, indicating restoration of antegrade flow to more than half of the affected vascular territory. In contrast, mTICI 2c–3 or mTICI 3 represent near-complete or complete reperfusion, thresholds that have been shown to correlate more strongly with favorable clinical outcomes. The expanded eTICI scale further refines the distinction between partial and near-complete reperfusion. Because these thresholds reflect progressively stricter definitions of angiographic success, comparisons across studies reporting different scales should be interpreted with caution.

## 4. Discussion

The seven included studies likely reflect the current real-world evidence base on periprocedural antiplatelet management during eCAS in acute TLs. Although highly heterogeneous with respect to study design, sample size, antiplatelet regimens, concomitant therapies (e.g., heparin, IVT), and outcome definitions, several consistent issues emerge regarding the safety and potential effectiveness of IV antiplatelet agents compared with conventional oral strategies such as ASA. Importantly, all included studies were observational. Consequently, the current evidence remains limited. Nevertheless, these studies provide valuable insight into evolving clinical practice.

Across the included studies, both GPIs and cangrelor were used as rapid-onset antiplatelet agents during eCAS. GPIs, particularly tirofiban and eptifibatide, were generally associated with favorable angiographic and functional outcomes [22,23]. Interestingly, tirofiban has been reported to be associated with improved clinical outcomes in patients with AIS due to large artery atherosclerosis who achieve complete reperfusion and present with high baseline National Institutes of Health Stroke Scale (NIHSS) scores, suggesting a potential benefit in this high-risk subgroup [27]. In contrast, abciximab was consistently associated with a higher incidence of sICH, particularly among older patients [25,26], suggesting an unfavorable safety profile in this setting.

In the ETIS Registry analyses [23,24], GPIs were independently associated with higher odds of functional independence compared with ASA. Similarly, in the ARTISTA Registry [22], tirofiban was associated with lower rates of in-stent thrombosis, reduced 24 h sICH, and improved recanalization compared with ASA. Collectively, these findings suggest that GPIs—particularly tirofiban—may offer improved technical and clinical outcomes without a clear signal of increased bleeding risk, aligning with prior observational evidence in AIS populations treated without MT [28].

Cangrelor, an intravenous P2Y_12_ inhibitor, is increasingly explored as an alternative to GPIs. Its proposed advantages include rapid and reversible platelet inhibition, limited renal dependence, and ease of transition to oral agents [29,30,31]. However, clinical data remain mixed. In a report by Delvoye et al., cangrelor was associated with numerically higher rates of favorable outcomes and lower mortality and sICH compared with abciximab [25]. Conversely, Pop et al. found that while cangrelor improved carotid stent patency at day 1, it did not translate into improved functional outcomes compared with ASA, and GPIs appeared to outperform cangrelor with respect to functional independence [23]. Jumaa et al. similarly observed that cangrelor use was associated with significantly higher rates of complete reperfusion, although this did not yield better functional outcomes [21]. Taken together, these findings suggest that cangrelor use is feasible and may be safe, with potential benefits for procedural success, but its superiority over GPIs or ASA in improving long-term clinical outcomes remains unproven.

Farooqui et al. provided a broader comparison of multiple antiplatelet strategies, reporting that both oral DAPT and IV regimens were associated with higher rates of successful and excellent reperfusion compared with no antiplatelet therapy, without an apparent increase in sICH risk [20]. Notably, cangrelor was associated with the highest odds of achieving excellent reperfusion, further supporting its potential role as a potent intraprocedural adjunct [20].

A central challenge in TL management is balancing carotid stent patency against hemorrhagic risk, particularly among patients treated with IVT. Reassuringly, none of the included studies demonstrated a statistically significant increase in sICH with either GPIs or cangrelor compared with ASA or other oral antiplatelet regimens. Jumaa et al. and Medina-Rodriguez et al. even suggested a trend toward lower hemorrhagic risk with tirofiban and cangrelor [21,22], although these findings should be interpreted cautiously given small sample sizes and heterogeneity in concomitant therapies, including IVT and periprocedural heparin use. Although white matter alterations (i.e., leukoaraiosis), especially moderate to severe, have been recognized as a significant risk factor for hemorrhagic transformation after reperfusion treatment in AIS, none of the studies included in our analysis reported on the prevalence and/or intensity of these lesions.

The included studies further suggest that although concurrent IVT represents an important confounder, modern IV antiplatelet agents such as cangrelor and low-dose tirofiban may maintain an acceptable safety profile even when used alongside thrombolytics. Available data also indicate substantial clinician-driven selection bias, as patients receiving IVT were generally less likely to receive more aggressive antiplatelet regimens. Nevertheless, our analysis suggests that these potent agents may improve reperfusion and stent patency without a statistically significant increase in sICH. In contrast, abciximab appears to carry a higher inherent hemorrhagic risk that may persist irrespective of prior IVT exposure [25,26]. Notably, a recent systematic review and meta-analysis including 24 studies evaluated the safety and efficacy of IVT + MT versus MT alone in AIS TL patients, and showed that the combination of IVT and MT did not increase the risk of sICH, even in the context of eCAS [32].

Procedural management of TLs typically involves either an antegrade (carotid-first) or retrograde (thrombectomy-first) strategy, with antegrade approaches more frequently reported in studies by Farooqui et al. [20], Marnat et al. [24], and Heck et al. [26]. While available data do not clearly support one sequence as uniformly superior, Medina-Rodriguez et al. reported that a retrograde strategy combined with early low-dose tirofiban initiation (after groin puncture and before angioplasty) was associated with significantly lower rates of in-stent thrombosis (1.3% vs. 9%) and sICH (3% vs. 12%) compared with ASA [22]. Collectively, these findings suggest that clinical success and safety may be influenced less by procedural sequence itself than by the timing and selection of short-acting IV antiplatelet agents, whereas abciximab appears to carry a higher intrinsic hemorrhagic risk irrespective of procedural approach [25,26]. Interestingly, in a recent multicenter registry study of eCAS in TL the timing of DAPT initiation following eCAS did not seem to modify the risk of late stent occlusion/restenosis, sICH, or the chance of achieving 90-day functional independence [33].

This analysis has several important limitations. Most notably, it is based on a narrative synthesis of exclusively observational studies. The current absence of published RCTs remains the major limitation of any literature review on this topic, although several trials are ongoing. Observational studies are inherently vulnerable to selection bias, particularly as antiplatelet choice often reflects operator preference, patient comorbidities, and concurrent use of thrombolytics. Furthermore, inconsistent definitions of outcomes (e.g., sICH, reperfusion success), lack of standardized dosing protocols, and heterogeneity in stenting techniques complicate direct comparisons. Importantly, only one included study (Marnat et al.) systematically evaluated antiplatelet efficacy using platelet function testing, with escalation to ticagrelor/prasugrel or repeat clopidogrel loading in non-responders [24].

Second, although GPIs were generally associated with favorable outcomes, they should not be regarded as a homogeneous drug class. Rather, they are clinically distinct agents with different pharmacokinetic and receptor-binding profiles, limiting the validity of class-wide conclusions and supporting the need for agent-specific evaluation.

Third, some studies included patients with isolated extracranial ICA occlusion alongside TL patients (Delvoye et al. [25]: 73% TL; Medina-Rodriguez et al. [22]: 82% TL), introducing case-mix dilution that may reduce generalizability to true TL AIS. Because isolated extracranial ICA occlusion represents a distinct pathophysiological and procedural entity—often differing in clot burden, recanalization complexity, stenting rates, and antiplatelet exposure—its inclusion may bias both efficacy (reperfusion, functional independence) and safety outcomes (e.g., sICH) in either direction. Overall, this heterogeneity may attenuate or misestimate TL-specific treatment effects and reduce the internal validity of conclusions intended to apply strictly to TL AIS.

Fourth, two of the seven included studies (Pop et al. [23], Marnat et al. [24]) were derived from the same ETIS Registry, with overlapping study periods and participating centers, raising the possibility of partial patient overlap. Such overlap violates the assumption of independence between datasets and may result in inadvertent “double counting,” disproportionately weighting registry-derived data within the pooled interpretation. Consequently, effect estimates may appear more precise than justified, heterogeneity may be underestimated, and overall conclusions may reflect registry-specific practice patterns rather than truly independent multicenter evidence sources.

**Figure 1:**
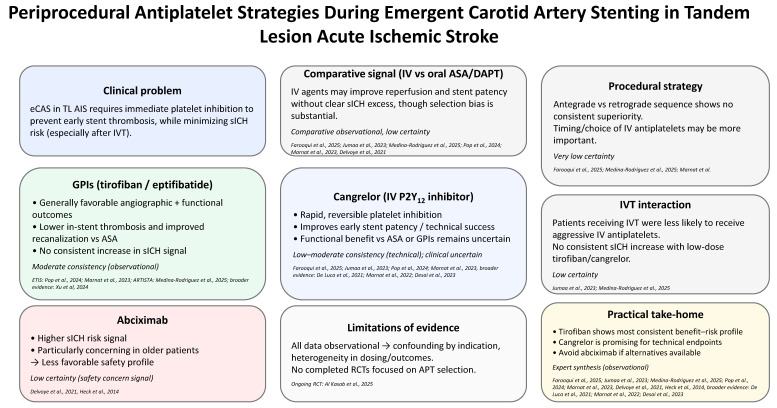
Periprocedural antiplatelet strategies during emergent carotid artery stenting in tandem lesion acute ischemic stroke. ASA: acetylsalicylic acid; DAPT: oral dual antiplatelet therapy; eCAS: emergent carotid artery stenting; GPI: glycoprotein IIb/IIIa inhibitors; IV: intravenous; IVT: intravenous thrombolysis; RCT; randomized controlled trial; sICH: symptomatic intracranial hemorrhage. Farooqui et al., 2025 [20]; Jumaa et al., 2023 [21]; Medina-Rodriguez et al., 2025 [22]; Pop et al., 2024 [23]; Marnat et al., 2023 [24]; Delvoye et al., 2021 [25]; Heck et al., 2015 [26]; Xu et al., 2024 [28]; De Luca et al., 2021 [29]; Marnat et al., 2022 [30]; Desai et al., 2023 [31]; Al Kasab et al., 2025 [34].

However, the principal strength of this narrative review lies in its inclusion and synthesis of available clinical data on periprocedural antiplatelet use in TL treatment from the contemporary study period. The main findings of our analysis are graphically depicted in Figure 1.

## 5. Conclusions and Future Perspectives

Based on the current evidence, GPIs—particularly tirofiban—appear to provide a favorable balance between efficacy and safety in maintaining carotid stent patency during eCAS for TLs [22,23,24]. Abciximab, in contrast, appears associated with increased sICH risk and may be less suitable for contemporary practice [25,26]. Cangrelor remains a promising alternative, offering rapid and reversible platelet inhibition, although robust comparative evidence remains limited [20,21,23,25,29,30,31].

Several ongoing RCTs are expected to address major evidence gaps in TL management, although most primarily compare endovascular strategies rather than antiplatelet regimens [3]. The PICASSO trial (Proximal Internal Carotid Artery Acute Stroke Secondary to Tandem or Local Occlusion Thrombectomy; NCT05611242) is currently the only trial noted to include antiplatelet subgroup analyses [34]. Future studies should directly compare IV antiplatelet agents within standardized protocols regarding dosing, timing, and procedural workflows, and with stratification by prior IVT exposure. Ideally, they should use randomized or adaptive trial designs. Incorporation of pharmacodynamic monitoring and cost-effectiveness analyses may further clarify optimal strategies, particularly given interindividual variability in response to clopidogrel [24]. Such pharmacodynamic endpoints may help explain variability in clinical response and should be incorporated more consistently into future studies.

Other possible lines of future research could focus on prospective studies of advanced imaging markers of infarct core/blood–brain barrier disruption. These could help identify subgroups most likely to benefit from intensified platelet inhibition while minimizing hemorrhagic risk. Finally, mechanistic and translational work focusing on early stent thrombosis biology and microvascular reperfusion (including no-reflow phenomena) may refine individualized antiplatelet selection during eCAS for TLs. Until such evidence becomes available, periprocedural antiplatelet selection should remain individualized, balancing hemorrhagic risk, procedural complexity, and institutional experience.

## Data Availability

No original dataset was generated.

## Supplementary Materials

The following supporting information can be downloaded at: https://www.mdpi.com/article/10.3390/jcm15093195/s1, Table S1: Procedural details of the included studies.

## Abbreviations

The following abbreviations are used in this manuscript:

ASA	Acetylsalicylic acid
sICH	Symptomatic intracranial hemorrhage
IAT	Intra-arterial thrombolysis
IVT	Intravenous thrombolysis
TL	Tandem lesion
GPI	Glycoprotein IIb/IIIa inhibitor
eCAS	Emergent carotid artery stenting
RCT	Randomized controlled trial
DAPT	Oral dual antiplatelet therapy
AIS	Acute ischemic stroke
MT	Mechanical thrombectomy
MCA	Middle cerebral artery
ICA	Internal carotid artery
ASPECTS	Alberta Stroke Program Early CT Score
CT	Computerized tomography
LVO	Large vessel occlusion
mRS	Modified Rankin Scale
NIHSS	National Institutes of Health Stroke Scale
