# Supplementary material for: Revisiting Antiplatelet Therapy in Acute Carotid Tandem Lesions
Source: J Clin Med. 2026 Apr 22;15(9):3195. doi: 10.3390/jcm15093195 (PMC13164354; doi:10.3390/jcm15093195)
Supplement: Supplementary file 1 [file jcm-15-03195-s001.zip › jcm-4270432-supplementary.pdf]

Supplementary table 1. Procedural details of the included studies

Study	Drug Dosage & Timing	Procedural Strategy	Stent Type
**Farooqui et al., 2025 [20]**	**Protocol*** **Cangrelor:** Bolus 15 μg/kg 2 min, followed by continuous infusion 2 μg/kg/min **Tirofiban:** 0.1 μg/kg/min infusion, no bolus **Eptifibatide:** 0.5 to 2 μg/kg/min infusion with or without 90 μg/kg bolus **ASA + clopidogrel:** ASA 325 mg or 300 mg IV# loading, maintenance 81 mg Clopidogrel 300 mg or 600 mg loading, maintenance 75 mg **ASA + ticagrelor:** ASA 325 mg loading, maintenance 81 mg Ticagrelor 180 mg loading, maintenance 90 mg *Based on the institutional protocol from the participating centers #Only used in the European participating center	**Antegrade** No APT: 68% SAPT: 79% DAPT: 68% IV APT: 72% p=0.221	Not specified
**Pop et al., 2025 [23]**	**ASA:** IV bolus 250–500 mg **Cangrelor full dose:** 30 μg/kg bolus + 4 μg/kg/min infusion (45%) + ASA (56%) **Cangrelor low dose:** 5 μg/kg bolus + 1 μg/kg/min infusion (55%) + ASA (18%) **GPIs:** either full dose or low dose (bolus+ infusion) according to IFU IV infusions stopped at the end of the procedure or continued up to the 24h follow-up imaging	Not specified	Not specified
**Marnat et al., 2025 [24]**	Intraoperative administration; ASA bolus, IV GPIs (abciximab, tirofiban, eptifibatide): bolus + continuous infusion $P2Y_{12}$ inhibitors: IV cangrelor (bolus + continuous infusion), oral clopidogrel or ticagrelor loading (intraprocedurally or immediately after, unable to swallow: through NGT) DAPT instituted after MT Clopidogrel – antiplatelet efficacy systematically tested (if inefficient – ticagrelor /prasugrel/new loading dose of clopidogrel)	**Antegrade** Aggressive APTs: 64% ASA-only: 59%, p=0.535	Not specified in main text
**Medina-Rodriguez et al., 2025 [22]**	IV infusion of APTs after groin puncture and before balloon angioplasty **IV ASA:** 250 mg bolus if concomitant IVT and/or prior oral ASA, otherwise 500 mg **IV tirofiban (low-dose):** 500 μg bolus + cont. inf. 200 μg/h 24 ± 2h **Heparin not used** After 20-24h: no ICH on CT: **ASA group**: ASA 100 mg PO + clopidogrel 300 mg PO loading **Tirofiban group**: ASA 300 mg + clopidogrel 300 mg PO loading **DAPT for 1 month**	**Retrograde** unless cervical ICA stenosis/occlusion prevented it	Wallstent
**Jumaa et al., 2023 [21]**	**No additional oral APTs** **Cangrelor:** 2 μg/kg/min with 15 μg/min bolus **Tirofiban:** 0.1 μg/kg/min infusion with no bolus **Eptifibatide:** 0.5–2 μg/kg/min infusion with or without 90 μg/kg bolus	Not specified	Not specified
**Delvoye et al., 2021 [25]**	**Abciximab:** 0.25 mg/kg IV bolus followed by continuous infusion 0.125 μg/kg/min for 12h **Cangrelor:** 30 μg/kg IV bolus followed by continuous infusion 4 μg/kg/min and discontinued at the end of the procedure **ASA:** 250 mg IV bolus **Heparin:** 50 IU/kg IV if no IVT	Not specified	**Abciximab:** Wallstent: 38%; Casper: 38%; Braided: 25%; FD: 25% **Cangrelor:** Wallstent: 67%; Casper: 22%; Braided: 11%; Open cells: 11% **ASA:** Wallstent:

			72%; Casper: 23%; FD: 2%; Open cells: 2%
**Heck et al, 2014 [26]**	**ASA: 300 mg** suppository on the table<br>**Abciximab:** loading dose in 12 patients (0.25 mg/kg just before stent placement, without preceding IVT) + in 1 patient after intraprocedural stent thrombosis<br>**Heparin:** 2,000 IU IV optionally<br>If no ICH: **clopidogrel** PO 600 mg loading at 24h, in patients with preceding IVT after 48h	**Antegrade**	Closed cell stent (XACT): 87%<br>Open cell stent (Precise): 13%

APT: antiplatelet; ASA: acetylsalicylic acid; CT: computerized tomography; DAPT: oral dual antiplatelet therapy; GPIs: glycoprotein IIb/IIIa inhibitors; ICH: intracranial hemorrhage; IFU: instructions for use; IU: international unit; IV: intravenous; IVT: intravenous thrombolysis; MT: mechanical thrombectomy; NGT: nasogastric tube; PO: peroral; SAPT: oral single antiplatelet therapy.
